# Thoracic paravertebral block with different doses of liposomal bupivacaine versus ropivacaine for postoperative analgesia in single-port thoracoscopic lung surgery: a randomized clinical trial

**DOI:** 10.1080/07853890.2026.2687941

**Published:** 2026-06-24

**Authors:** Liyuan Ren, Zhenhua Nan, Yanshuang Li, Yanping Wang

**Affiliations:** Department of Anesthesiology, Pain and Perioperative Medicine, The First Affiliated Hospital of Zhengzhou University, Zhengzhou, Henan, China

**Keywords:** Liposomal bupivacaine, ropivacaine, thoracic paravertebral block, pain

## Abstract

**Objective:**

To evaluate the analgesic efficacy of thoracic paravertebral block (TPVB) with different doses of liposomal bupivacaine (LB) or ropivacaine in patients undergoing single-port thoracoscopic lung surgery.

**Methods:**

A total of 105 patients scheduled for video-assisted single-port thoracoscopic lung surgery were randomized in a 1:1:1 ratio into three groups: low-dose LB group (group LL), high-dose LB group (group HL), or ropivacaine group (group R). All received ultrasound-guided TPVB at the T5/6 level preoperatively. The primary outcome was the area under the curve (AUC) of NRS of pain at activity (AUC-aNRS) from 1 to 72 h postoperatively. Secondary outcomes included the AUC of NRS of pain at rest (AUC-rNRS) from 1 to 72 h postoperatively, NRS of pain at rest and at activity at 1, 6, 24, 48, and 72 h postoperatively, and the cumulative opioid consumption at 24, 48, and 72 h postoperatively. Additionally, postoperative recovery and adverse events were assessed.

**Results:**

AUC-aNRS differed significantly among groups (*p* = 0.0092), with high-dose LB lower than low-dose LB (*p* = 0.0071), but not versus ropivacaine. No significant difference was found in AUC-rNRS (*p* > 0.05). The group-by-time interactions for NRS of pain at rest and at activity were not significant (*p* > 0.05). Cumulative opioid consumption at 24, 48, and 72 h was lower in group HL versus group LL (all *p* < 0.017), but not versus ropivacaine. Postoperative recovery and adverse events showed no differences (*p* > 0.05).

**Conclusion:**

LB combined with TPVB is not superior to ropivacaine for postoperative analgesia in single-port thoracoscopic lung resection.

## KEY MESSAGES

1. This study is the first to compare the analgesic efficacy of TPVB combined with two doses of LB and traditional ropivacaine in patients undergoing thoracoscopic lung surgery.
2. Within 72 hours postoperatively, neither low-dose nor high-dose LB showed superiority over ropivacaine in reducing NRS of pain or opioid use. High-dose LB showed dose-dependent analgesic and opioid-sparing effects, though the pain reduction was clinically limited.
3. Single-point TPVB with LB alone may not be an effective alternative to ropivacaine, optimizing strategies (e.g. ultrasound-guided multi-point injection, combining with plain bupivacaine) are needed to improve LB’s efficacy in TPVB in the future.

## 1. Introduction

Video-assisted thoracoscopic surgery (VATS) is a minimally invasive procedure widely used in thoracic surgery, offering advantages such as minimal trauma, less bleeding, and faster recovery [1,2]. Despite these advantages, patients often experience significant acute and chronic postoperative pain, which can impede the rehabilitation process [3,4]. Therefore, optimizing postoperative analgesia for VATS remains an important challenge [5]. Multimodal analgesia strategies, which combine analgesic methods with different mechanisms of action, can enhance pain relief while reducing opioid consumption and related side effects, thereby facilitating enhanced recovery after surgery. Among them, regional nerve block is a key component of perioperative multimodal analgesia [6,7].

Thoracic paravertebral block (TPVB) is a first-line regional block method for postoperative analgesia in thoracic surgery. Through an injection of local anesthetic into the thoracic paravertebral space, it can produce ipsilateral multi-segment somatic and sympathetic nerve blockade. Its analgesic effect is comparable to that of epidural block, while it has fewer complications [6,8,9]. Currently, ropivacaine (0.25%–0.5%, 20–30 mL) is widely used as the local anesthetic for TPVB. Although it effectively alleviates early postoperative pain and reduces opioid requirements, its duration of action is relatively short (typically 6–12 h), making it difficult to fully cover the peak postoperative pain period.

Liposomal bupivacaine (LB) is a long-acting local anesthetic formulation. By utilizing multivesicular liposome technology, it enables the sustained release of bupivacaine, maintains local drug concentrations at the administration site, and reduces systemic absorption – thereby extending the analgesic duration to 48–72 h [10,11]. Since it can avoid complications associated with perineural catheters, including infection, leakage, catheter displacement, and a high failure rate, LB combined with peripheral nerve block is regarded as a potential alternative [12]. However, current clinical evidence regarding the analgesic effect of TPVB with different doses of LB for single-port thoracoscopic lung surgery remains relatively scarce. Therefore, this study aims to evaluate the effect of TPVB with two doses of LB (low dose: 133 mg; high dose: 266 mg) or ropivacaine on postoperative pain control after VATS through a prospective controlled clinical trial. This study hypothesizes that, compared with ropivacaine, LB combined with TPVB can reduce the area under the curve of the NRS of pain at activity (AUC-aNRS) from 1 to 72 h postoperatively, and high-dose LB is superior to low-dose LB in analgesic efficacy.

## 2. Materials and methods

### 2.1. Study design and ethics

This investigation was designed as a single-center, prospective, randomized, double-blind, controlled clinical trial. Ethical approval was granted by the Scientific Research and Clinical Trial Ethics Committee of the First Affiliated Hospital of Zhengzhou University on December 19, 2024 (approval number: 2024-KY-2120-001), and the trial was registered in the Chinese Clinical Trials Registry under the identifier ChiCTR2500102806 on May 20, 2025. Written informed consent was acquired from all participants prior to study commencement. This study was conducted in full compliance with the Declaration of Helsinki, and all procedures performed on human subjects were reviewed and approved by the pertinent ethics review committee. Furthermore, the reporting of this trial adhered strictly to the Consolidated Standards of Reporting Trials (CONSORT) checklist.

### 2.2. Participants

Patients aged 18 to 65 years, with American Society of Anesthesiologists (ASA) physical status I-III, scheduled for video-assisted single-port thoracoscopic lung surgery under general anesthesia at the First Affiliated Hospital of Zhengzhou University from May 2025 to August 2025, were recruited. Exclusion Criteria: previous same-side surgery; diagnosed mental illness; history of drug or alcohol abuse; body mass index (BMI) < 18 kg/m^2^ or > 30 kg/m^2^; severe hepatic or renal dysfunction; coagulation disorders; puncture site infections; long-term use of chronic analgesic drugs; spinal deformities; communication difficulties. Withdrawal criteria included intraoperative conversion to open surgery, serious adverse reactions during or after surgery, postoperative transfer to the ICU, and patients or family members requesting to withdraw midway.

### 2.3. Randomization and blinding

Using a computer-generated randomization schedule, patients were randomized in a 1:1:1 ratio to three groups: the low-dose LB group (group LL), the high-dose LB group (group HL), and the ropivacaine group (group R). All group allocations were sealed in sequentially numbered opaque envelopes to ensure concealment; these envelopes were opened by a nurse in the anesthesia preparation room, who then prepared and dispensed the investigational drugs. Specifically, group LL received 133 mg LB diluted to 20 mL with saline; group HL received 266 mg LB (20 mL stock solution); and group R received 66 mg ropivacaine diluted to 20 mL with saline. In the anesthesia preparation room, all patients underwent ultrasound-guided TPVB performed by the same experienced anesthesiologist, while intraoperative anesthesia was administered by another anesthesiologist. The patients, intraoperative anesthesiologist, postoperative follow-up physician, and surgeon were blinded to the group assignment. Due to the inherent color difference between the study drugs (LB is milky white while ropivacaine is clear), the anesthesiologist performing TPVB could not be blinded to the group assignment; this physician was only responsible for the block procedure and did not participate in any other study-related processes, including patient enrollment, randomization, intraoperative anesthesia management, postoperative follow-up, or statistical analysis. Blinding of patients was effectively achieved because the TPVB was performed with patients in the lateral decubitus position with the injection site on their back, making it impossible for patients to observe the drug color or injection process, and the performing anesthesiologist did not disclose the type of study drug to any patient. Blinding was maintained until the completion of all statistical analyses of the study data.

### 2.4. Intervention

In the anesthesia preparation room, peripheral venous access was established in the upper limb, and the patient was placed in a lateral decubitus position for TPVB. Under real-time ultrasound guidance, using an in-plane approach, place the low-frequency convex array probe at the T5/6 level, approximately 2–3 cm from the midline of the spine. Clearly identify key structures such as the transverse process, hyperechoic costotransverse ligament, and the underlying pleura. Insert the puncture needle along the long axis of the probe from lateral to medial, penetrate the costotransverse ligament, and enter the paravertebral space of the thoracic vertebrae. After confirming the needle tip position and aspirating negatively to ensure no blood return, the local anesthetic was injected slowly. Downward displacement of the pleura indicated a successful block. The same anesthesiologist performed all TPVB. Low-dose LB group: 133 mg of LB (20 mL, 0.67%) was injected into the paravertebral space. High-dose LB group: 266 mg of LB (20 mL, 1.33%) was injected into the paravertebral space. Ropivacaine group: 66 mg of ropivacaine (20 mL, 0.33%) was injected into the paravertebral space. Thirty minutes after the completion of the block procedure, the sensory block effect was evaluated using the pinprick test. The block was considered effective when the patient reported a reduction in pain sensation.

### 2.5. Outcomes

The primary outcome was the AUC-aNRS from 1 to 72 h postoperatively.

The secondary outcomes included: the AUC of the NRS of pain at rest (AUC-rNRS) from 1 to 72 h postoperatively; NRS of pain at rest and activity (cough) at 1, 6, 24, 48, and 72 h postoperatively; cumulative opioid consumption (converted to morphine equivalents) at 24, 48, and 72 h postoperatively; incidence of moderate-to-severe pain at activity (NRS > 3) within 72 h postoperatively; time to removal of chest drain; length of hospitalization; block-related adverse events (hypotension, pneumothorax, vascular injury, local anesthetic toxicity, high epidural anesthesia, total spinal anesthesia, spinal cord injury, etc.); and other adverse events (nausea, vomiting, dizziness, fever, itching, constipation, urinary retention, arrhythmia, etc.).

### 2.6. Anesthetic and surgical management

The patient was fasted for 8 h and clear fluids for 2 h preoperatively. Standard monitoring was applied in the operating room, including ECG, NBP, SpO_2_. An arterial catheter was placed under local anesthesia for invasive blood pressure monitoring. Anesthesia was induced with propofol (1.5–2 mg/kg), sufentanil (0.6 ug/kg), and rocuronium (0.6 mg/kg). Following induction, an endobronchial tube was placed, and mechanical ventilation was initiated with the following settings: tidal volume 4–6 mL/kg, respiratory rate 12–20 beats/min, oxygen flow 2 L/min, FiO_2_ 100%, and end-tidal CO_2_ maintained at 35–40 mmHg. Anesthesia was maintained with sevoflurane (1%–2%), propofol infusion (1.5–2 mg/kg/h), and remifentanil infusion (0.1–0.3 ug/kg/min). Rocuronium (0.3 mg/kg) was administered intermittently. The remifentanil infusion rate was titrated according to vital signs, surgical progress, and stimulation intensity. At skin closure, oxycodone (0.08 mg/kg) and palonosetron (0.25 mg) were administered for postoperative analgesia and postoperative nausea and vomiting prophylaxis. Upon completion of surgery, sevoflurane and remifentanil were discontinued, and a patient-controlled intravenous analgesia (PCIA) pump was connected. The PCIA solution contained hydromorphone (0.2 mg/kg) and palonosetron (0.25 mg) in 200 mL of normal saline, with a background infusion of 1 mL/h, a bolus dose of 4 mL, and a lockout time of 10 min. Rescue analgesia with intravenous oxycodone (3 mg) was administered if the NRS of pain remained > 3 after three consecutive PCIA demands.

### 2.7. Sample size calculation and statistical analysis

The sample size was estimated using pilot study data, with 10 patients enrolled in each of the three groups. The mean ± SD of AUC‑aNRS in the three groups were 190.3 ± 24.2 (group R), 209.4 ± 25.2 (group LL), and 186.9 ± 27.0 (group HL). Using one‑way ANOVA to compare the overall differences among the three groups (α = 0.05, power = 0.90, pooled SD = 24.7), 28 participants per group were required. Accounting for a 15% dropout rate and taking a conservative rounded number, 35 participants were actually enrolled in each group, totaling 105 participants.

All statistical analyses and figure generation were carried out with SPSS 27.0 and GraphPad Prism 10.1. Normality of variable distribution was verified *via* the Shapiro-Wilk test. Normally distributed data were summarized as mean ± standard deviation (x ± s) and compared among groups using one-way ANOVA. Non-normally distributed data were described as median (interquartile range) [M (Q1, Q3)], with intergroup differences analyzed by the Kruskal–Wallis H test. Categorical data were presented as n (%), and intergroup comparisons were performed using the χ^2^ test or Fisher’s exact test. *p* < 0.05 was defined as statistically significant.

The AUC of NRS of pain from 1 to 72 h postoperatively among the three groups was compared using one-way ANOVA. When the overall ANOVA was significant, post-hoc pairwise comparisons were performed with Bonferroni correction, and the corrected significance level was set at α’ = 0.05/3 ≈ 0.017. NRS of pain at different time points were analyzed using linear mixed‑effects models (LMM) with restricted maximum likelihood (REML) estimation. The models included time, group, and the time × group interaction as fixed effects and a random intercept for each patient. When the time × group interaction was statistically significant, simple effect analyses were performed. To control the overall type I error, the Bonferroni correction was applied, and the significance level was set at α’ = 0.05/(5 × 3) ≈ 0.0033. The Kruskal–Wallis H test was used to compare cumulative opioid consumption among the three groups at each time point (24, 48, and 72 h postoperatively), when significant, pairwise comparisons were performed with Bonferroni correction (α’ = 0.05/3 ≈ 0.017). All secondary outcomes were considered exploratory.

## 3. Results

**Figure 1.**
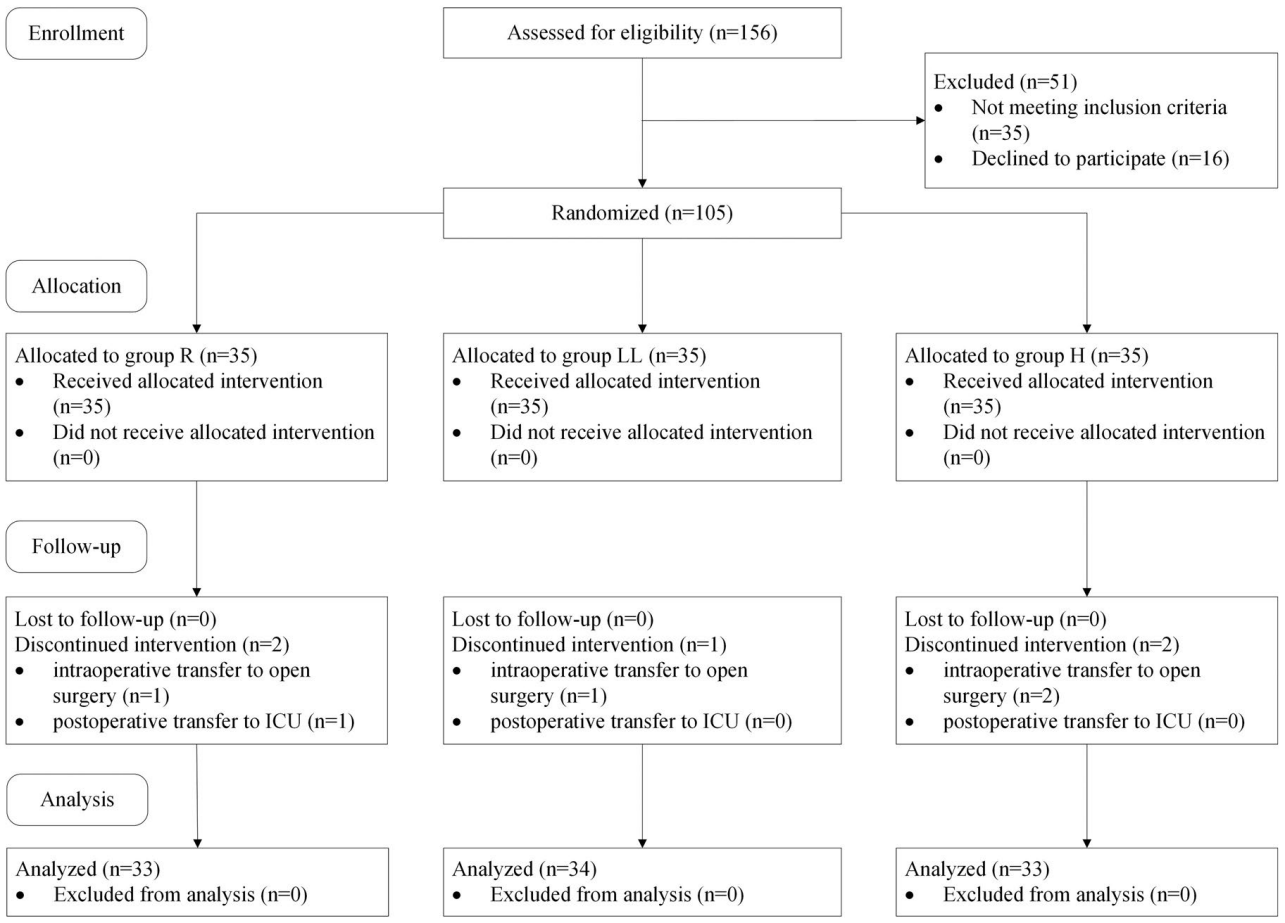
Study flow chart. Abbreviations: group R, ropivacaine group; group LL, low-dose liposomal bupivacaine group; group HL, high-dose liposomal bupivacaine group.

A total of 156 patients were screened for eligibility, of whom 51 were excluded before randomization. The remaining 105 patients were enrolled and randomly assigned to three groups: group R (*n* = 35), group LL (*n* = 35), and group HL (*n* = 35). One patient from group R, one from group LL, and two from group HL were excluded due to conversion to open surgery during surgery. Additionally, one patient from group R was excluded due to postoperative transfer to the ICU. Ultimately, a total of 100 patients (33 in group R, 34 in group LL, and 33 in group HL) were included in the final analysis. The study process is illustrated in Figure 1.

### 3.1. Baseline and intraoperative characteristics

The baseline characteristics were well-balanced among the three groups (Table 1). There were no significant differences among the three groups in terms of surgical type, duration of surgery, duration of anesthesia, infusion volume, bleeding volume, urine volume, or remifentanil consumption (*p* > 0.05) (Table 2).

**Table 1.** Baseline characteristics.

Variable	Group R (*n* = 33)	Group LL (*n* = 34)	Group HL (*n* = 33)	*P*
Age, yr	55.0 (44.5–61.0)	54.0 (42.0–62.0)	52.0 (46.5–58.5)	0.834
Male, n (%)	15 (45.5)	18 (52.9)	16 (48.5)	0.827
BMI, kg/m^2^	23.9 ± 2.6	23.7 ± 2.4	23.2 ± 2.7	0.531
ASA classification, n (%)				0.987
I	5 (15.2)	7 (20.6)	6 (18.2)	
II	26 (78.8)	25 (73.5)	25 (75.8)	
III	2 (6.1)	2 (5.9)	2 (6.1)	
Smoker, n (%)	6 (18.2)	8 (23.5)	5 (15.2)	0.675
Hypertension, n (%)	11 (33.3)	9 (26.5)	8 (24.2)	0.692
Diabetes, n (%)	3 (9.1)	4 (11.8)	4 (12.1)	0.911
aCCI	4.0 (3.0–5.0)	4.0 (2.0–5.0)	4.0 (3.0–4.5)	0.914
Preoperative 1-day NRS of pain	0 (0–0)	0 (0–0)	0 (0–0)	0.932

Notes: data are expressed by median (interquartile range), n (%), or mean ± standard deviation.

Abbreviations: group R, ropivacaine group; group LL, low-dose liposomal bupivacaine group; group HL, high-dose liposomal bupivacaine group; BMI, body mass index; ASA, American Society of Anesthesiologists; aCCI, age-adjusted Charlson comorbidity index; NRS, numeric rating scale.

**Table 2.** Intraoperative data.

Variable	Group R (*n* = 33)	Group LL (*n* = 34)	Group HL (*n* = 33)	*P*
Type of surgery, n (%)				0.935
Wedge resection of the lung	13 (39.4)	11 (32.4)	11 (33.3)	
Segmentectomy of the lung	14 (42.4)	18 (52.9)	16 (48.5)	
Lobectomy of the lung	6 (18.2)	5 (14.7)	6 (18.2)	
Duration of surgery (min)	102.0 (57.5–139.0)	109.0 (60.8–157.3)	103.0 (74.0–149.5)	0.420
Duration of anesthesia (min)	125.0 (83.0–166.5)	138.0 (79.8–182.0)	131.0 (97.5–178.5)	0.495
Infusion volume (mL)	800.0 (500.0–1100.0)	900.0 (575.0–1400.0)	900.0 (600.0–1300.0)	0.427
Bleeding volume (mL)	50.0 (20.0–50.0)	50.0 (27.5–57.5)	50.0 (30.0–60.0)	0.619
Urine volume (mL)	300.0 (200.0–600.0)	350.0 (250.0–650.0)	350.0 (200.0–600.0)	0.548
Remifentanil consumption (mg)	1.3 (0.8–1.8)	1.4 (0.9–1.9)	1.1 (0.9–1.8)	0.870

Notes: data are expressed by n (%) or median (interquartile range).

Abbreviations: group R, ropivacaine group; group LL, low-dose liposomal bupivacaine group; group HL, high-dose liposomal bupivacaine group.

### 3.2. Primary outcomes and key secondary outcomes

**Figure 2.**
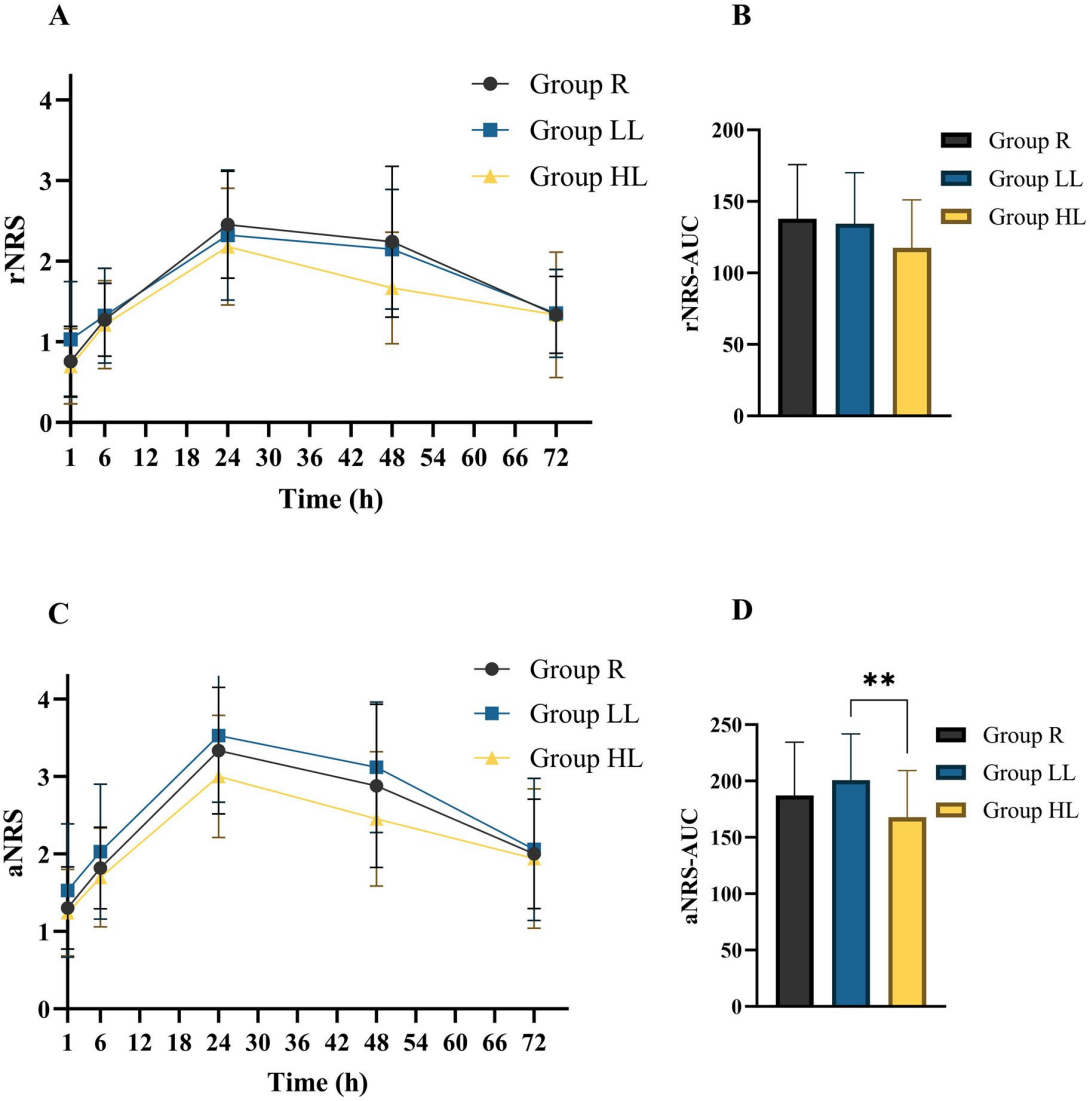
NRS of pain at different time points and AUC of NRS. Notes: (a) rNRS at different time points; (b) AUC of rNRS; (c) aNRS at different time points; (d) AUC of aNRS. Data are expressed by mean ± standard deviation, ** *P* < 0.017 (Bonferroni-corrected significance level) vs group LL. Abbreviations: group R, ropivacaine group; group LL, low-dose liposomal bupivacaine group; group HL, high-dose liposomal bupivacaine group; NRS, numeric rating scale; rNRS, NRS of pain at rest; aNRS, NRS of pain at activity; AUC, area under the curve.

A significant difference was found in the AUC-aNRS from 1 to 72 h postoperatively among the three groups (*p* = 0.0092), where the AUC-aNRS in group HL (167.8 ± 22.6) was significantly lower than that in group LL (200.8 ± 23.7) (*p* = 0.0071), but no significant difference was found compared to group R (187.3 ± 23.8) (*p* > 0.017) (Figure 2). There was no significant difference in the AUC-rNRS among the three groups (*p* > 0.05) (Figure 2).

NRS of pain at rest and at activity at 1, 6, 24, 48, and 72 h postoperatively were analyzed using LMM. The results showed that the group‑by‑time interaction was not statistically significant for rNRS (*F* = 1.495, *p* = 0.156) nor for aNRS (*F* = 0.796, *p* = 0.607), indicating that the trajectories of pain over 72 h did not differ among the three groups. Therefore, no post‑hoc pairwise comparisons were performed across groups at individual time points. Descriptive data of NRS of pain at each time point are presented in Table 3.

**Table 3.** NRS Of pain at different time points.

Variable	Group R (*n* = 33)	Group LL (*n* = 34)	Group HL (*n* = 33)
NRS of pain at rest			
T_1h_	0.8 ± 0.4	1.0 ± 0.7	0.7 ± 0.5
T_6h_	1.3 ± 0.5	1.3 ± 0.6	1.2 ± 0.5
T_24h_	2.5 ± 0.7	2.3 ± 0.8	2.2 ± 0.7
T_48h_	2.2 ± 0.9	2.2 ± 0.7	1.7 ± 0.7
T_72h_	1.3 ± 0.5	1.4 ± 0.5	1.3 ± 0.8
NRS of pain at activity			
T_1h_	1.3 ± 0.5	1.5 ± 0.9	1.2 ± 0.6
T_6h_	1.8 ± 0.5	2.0 ± 0.9	1.7 ± 0.6
T_24h_	3.3 ± 0.8	3.5 ± 0.9	3.0 ± 0.8
T_48h_	2.9 ± 1.1	3.1 ± 0.8	2.5 ± 0.9
T_72h_	2.0 ± 0.7	2.1 ± 0.9	1.9 ± 0.9

Notes: data are expressed by mean ± standard deviation.

Abbreviations: NRS, numeric rating scale; group R, ropivacaine group; group LL, low-dose liposomal bupivacaine group; group HL, high-dose liposomal bupivacaine group.

### 3.3. Secondary outcomes about cumulative opioid consumption

Significant differences were observed in cumulative opioid consumption at 24, 48, and 72 h postoperatively among the three groups (*p* < 0.05). The cumulative opioid consumption at these time points in group HL was lower than that in group LL (*p* = 0.008, *p* = 0.006, *p* = 0.005, respectively), but not significantly different from group R (*p* > 0.017) (Table 4).

**Table 4.** Cumulative opioid consumption at different time points (mg).

Variable	Group R (*n* = 33)	Group LL (*n* = 34)	Group HL (*n* = 33)	*P*
T_24 h_	13.9 (12.1–15.6)	14.9 (13.0–16.9)	12.8 (11.5–14.9)^#^	0.029
T_48 h_	27.8 (26.0–32.6)	29.9 (27.6–38.8)	26.1 (22.3–31.6)^#^	0.023
T_72 h_	39.9 (36.7–46.8)	43.7 (38.2–57.6)	37.3 (32.6–45.0)^#^	0.019

Notes: data are expressed by median (interquartile range).

^#^ *p* < 0.017 (Bonferroni-corrected significance level) vs group LL.

Abbreviations: group R, ropivacaine group; group LL, low-dose liposomal bupivacaine group; group HL, high-dose liposomal bupivacaine group.

### 3.4. Secondary outcomes about postoperative recovery

No significant differences were observed among the three groups in terms of extubation time, time in the PACU, time to removal of chest drain, incidence of moderate-to-severe pain at activity within 72 h postoperatively, or length of hospitalization (*p* > 0.05) (Table 5).

**Table 5.** Postoperative recovery.

Variable	Group R (*n* = 33)	Group LL (*n* = 34)	Group HL (*n* = 33)	*P*
Extubation time (min)	22.0 (19.0–24.5)	23.0 (18.0–24.5)	21.0 (18.0–23.5)	0.411
Time in the PACU (min)	54.0 (48.0–60.5)	56.0 (48.5–64.3)	54.0 (49.5–62.0)	0.753
Time to removal of chest drain (h)	74.0 (71.0–87.0)	75.0 (68.8–83.8)	73.0 (67.5–84.5)	0.583
Moderate-to-severe pain within 72 h at activity postoperatively, n (%)	13 (39.4)	14 (41.2)	9 (27.3)	0.438
Length of hospitalization (d)	5.0 (5.0–6.0)	5.0 (5.0–6.0)	5.0 (5.0–6.0)	0.819

Notes: data are expressed by median (interquartile range) or n (%).

Abbreviations: group R, ropivacaine group; group LL, low-dose liposomal bupivacaine group; group HL, high-dose liposomal bupivacaine group; PACU, postanesthesia care unit.

### 3.5. Adverse events

No block-related complications or arrhythmia occurred in any of the groups. There were no significant differences among the three groups in the incidence of adverse events such as nausea and vomiting, dizziness, fever, pruritus, constipation, or urinary retention (*p* > 0.05) (Table 6).

**Table 6.** Adverse events.

Variable	Group R (*n* = 33)	Group LL (*n* = 34)	Group HL (*n* = 33)	*P*
Block-related adverse events	0 (0)	0 (0)	0 (0)	1.000
Other adverse events				
Nausea and vomiting	5 (15.2)	7 (20.6)	5 (15.2)	0.791
Dizziness	5 (15.2)	5 (14.7)	4 (12.1)	0.929
Fever	2 (6.1)	2 (5.9)	2 (6.1)	0.999
Pruritus	1 (3.0)	1 (2.9)	0	0.605
Constipation	2 (6.1)	2 (5.9)	1 (3.0)	0.817
Urinary retention	0	0	0	1.000
Arrhythmia	0	0	0	1.000

Notes: data are expressed by n (%).

Abbreviations: group R, ropivacaine group; group LL, low-dose liposomal bupivacaine group; group HL, high-dose liposomal bupivacaine group.

## 4. Discussion

This study is the first to compare ropivacaine with two distinct doses of LB for TPVB in patients undergoing single-port thoracoscopic lung resection. The results demonstrated that within 72 h postoperatively, neither the low-dose (133 mg) nor the high-dose (266 mg) LB group showed significant differences in the AUC of NRS from 1 to 72 h postoperatively or opioid consumption compared to the ropivacaine group. However, the high-dose LB group exhibited some advantages over the low-dose LB group in the AUC-aNRS and opioid sparing, though the pain reduction was clinically limited.

Since being approved by the U.S. FDA for local infiltration analgesia in 2011, the indications for LB have been expanded to several peripheral nerve blocks, including interscalene brachial plexus block, popliteal sciatic nerve block, and adductor canal block. Additionally, studies have investigated its potential application in supraclavicular brachial plexus block [13], transversus abdominis plane block [14,15], intercostal nerve block [16,17], serratus anterior plane block [18,19], erector spinae plane block [20,21]. However, the clinical efficacy of LB in peripheral nerve blocks remains controversial. A narrative review by Ilfeld et al. pointed out that in 92% of the relevant studies, the analgesic effect of plain bupivacaine (PB) was superior to that of LB. They argued that current evidence does not support the routine use of LB in peripheral nerve blocks, and suggested that future studies should focus on optimizing injection techniques (e.g. precise perineural injection), combination therapies (e.g. co-administration with PB), clarifying dose-effect relationships, and conducting real-world cost-effectiveness analyses [22]. Similarly, a study by Hussain et al. indicated that LB only provides statistically significant but not clinically significant improvements in analgesia in peripheral nerve blocks, and its purported advantages are more frequently observed in industry-sponsored trials. High-quality evidence does not support LB as a replacement for traditional local anesthetics (e.g. PB, ropivacaine) [23]. A study on ulnar nerve block in healthy volunteers further showed that the success rate of sensory block with LB was significantly lower than that with PB (32% vs 100%), with slower onset, shorter duration, and unpredictable residual block. In that study, Zadrazil et al. compared 13.3 mg of LB with 15 mg of PB – doses that were chemically equivalent but not clinically equivalent [24].

Notably, despite ongoing controversies regarding the use of LB in peripheral nerve blocks, clinical evidence supporting the use of LB combined with thoracic TPVB for analgesia in thoracoscopic lung surgery remains relatively limited. Furthermore, no existing studies have systematically investigated how varying LB doses affect analgesic efficacy. To address these knowledge gaps, the present study was specifically designed to compare two doses of LB (133 mg and 266 mg) with ropivacaine, with the primary objective of defining the actual analgesic value of LB combined with TPVB in patients undergoing single-port thoracoscopic lung resection. Consistent with the aforementioned findings, our study demonstrated that, for pain management in thoracoscopic lung resection, LB combined with single-point TPVB was not superior to ropivacaine in terms of NRS of pain or opioid consumption. In contrast, a recently published study by Shang et al. demonstrated that TPVB at the T5/6 level with LB (20 mL, 177 mg) significantly alleviated acute pain in patients undergoing video-assisted thoracoscopic lung resection. AUC of NRS pain scores within 72 h postoperatively was significantly lower in the LB group than in the bupivacaine group [25]. After a careful comparative analysis, we attribute the inconsistent results to the following three main factors: (1) This study focused exclusively on single-port video-assisted thoracoscopic lung resection, a more minimally invasive procedure associated with relatively mild overall postoperative pain intensity. As a result, LB lacked sufficient room to demonstrate its analgesic advantages. (2) The control group in this study received 0.33% ropivacaine, which has a longer duration of analgesia and more stable efficacy compared to 0.25% bupivacaine [26]. This may have further narrowed the relative advantage of LB over the control group, making it difficult to observe the analgesic benefits of LB. (3) Although there was a trend toward reduced pain scores in the high-dose LB group compared to the ropivacaine group, the difference did not reach statistical significance. This may be due to insufficient statistical power resulting from the relatively small sample size, and further validation through large-sample studies is required. In addition, in this study, although the AUC-aNRS in the high-dose group was significantly lower than that in the low-dose group, showing a statistical difference, the differences in pain scores between the two groups at all postoperative time points were less than 1 point, failing to reach the well-recognized Minimal Clinically Important Difference (MCID) for pain score improvement (1 point) [27]. Thus, the clinical benefits remain limited.

Recently, a series of studies have suggested that LB combined with multi-point TPVB may provide limited clinical benefits. In a study by Wu et al. investigating analgesia for the Nuss procedure, LB combined with TPVB (block level: T4/5–T7/8, two puncture points per side adjusted based on sternal depression position; 0.67% LB or 0.25% PB, 4–8 mL per point) reduced postoperative opioid consumption and the incidence of rebound pain compared with PB, but did not demonstrate significant advantages in pain NRS or control of moderate-to-severe pain [28]. Similarly, Wang et al. reported that LB combined with TPVB (block level: T7/8–T10/11, four puncture points, 7.5 mL per point; LB 4.43 mg/mL or PB 5 mg/mL) significantly reduced postoperative opioid consumption and prolonged the time to first opioid use in patients undergoing hepatectomy compared with PB-dexamethasone, but no significant differences were observed in pain NRS, inflammatory markers, or length of hospital stay [29]. Yang et al. compared LB and PB combined with TPVB in patients undergoing biportal VATS, injecting 10 mL (133 mg) of LB or 10 mL (18.75 mg) of PB into the T4/5 and T7/8 interspaces, respectively. Their results showed that LB prolonged the time to first postoperative analgesic request and improved the NRS of pain at rest; although it slightly reduced postoperative opioid consumption, this difference was not statistically significant. Additionally, LB had no significant effect on cough-related pain NRS. Its role in thoracic regional anesthesia thus requires further validation [30].

Compared with the aforementioned studies, neither dose of LB combined with TPVB in our study demonstrated analgesic advantages. An important factor contributing to this discrepancy may be the use of different injection techniques. We noticed that multi-point injection was adopted in the aforementioned studies to expand the block range, while only a single injection at the T5/6 level was used in this study. Owing to the large particle size of LB liposomes, their tissue diffusion capacity is relatively poor. In the structurally dense paravertebral space, LB is more likely to be retained near the injection site, making it difficult to extensively cover multiple spinal segments. The single-point injection technique further restricts drug distribution, which may significantly impair block efficacy. In addition, several other factors may also jointly contribute to the suboptimal results of this study: (1) The initial free drug concentration of LB is significantly low, with only approximately 3% of bupivacaine existing in the free state. In our study, the initial free concentrations of bupivacaine in the low-dose and high-dose LB groups were only approximately 0.02% and 0.04%, respectively – far lower than the effective block concentration of ropivacaine in the control group. This likely prevented the formation of an effective nerve block following injection. (2) Although LB possesses the ‘bimodal release’ characteristic, its sustained release rate is relatively low and unstable [31,32]. Studies have shown that infusing 0.125% bupivacaine at a rate of 5 mL/h can provide sufficient free drug for nerve blocking: approximately 6.3 mg/h. According to the calculation by Orebaugh et al. even when the maximum approved dose of LB (266 mg) is used, it can only provide about 2.6 mg of free bupivacaine per hour, which is still lower than the effective dose required for conventional regional blocks [33]. (3) The paravertebral space is rich in blood supply, which may accelerate the systemic absorption and clearance of bupivacaine, thereby further reducing the local drug concentration. As a result, the actual duration and intensity of the block are not as expected. (4) The sources of acute pain after VATS are complex, including incisional pain (involving the skin, muscles, and bones), visceral pain (affecting the lungs and mediastinum), stimulation from thoracic drainage tubes, inflammatory pain, and so on [34]. TPVB mainly acts on somatic pain, but its ability to control visceral pain and pain related to drainage tube irritation is limited. If the patient’s pain mainly stems from the latter, the overall analgesic effect of LB will be greatly reduced even if it can prolong the duration of nerve block.

This study has several limitations. First, it is a single‑center, small‑sample study. The sample size was calculated to detect an overall difference among the three groups. Therefore, the statistical power for individual pairwise comparisons (e.g. high‑dose LB group vs. control, low‑dose LB group vs. control, and high‑dose LB group vs. low‑dose LB group) is insufficient, and these pairwise comparisons should be interpreted as exploratory rather than confirmatory. Future multicenter, large‑sample randomized controlled trials are needed to validate our findings. Second, TPVB was performed *via* a single-point injection (at the T5/6 level). This administration method restricted the drug diffusion range within the paravertebral space, thereby compromising the final block efficacy. Third, no objective assessments were conducted on the onset time, block plane range, or regression process of sensory block, which prevented the accurate determination of the actual block range and quality. Fourth, the anesthesiologist performing the TPVB could not be blinded due to the color difference between the study drugs, which might introduce performance bias. However, this anesthesiologist did not participate in other study procedures, the procedure was strictly standardized, and both patients and outcome assessors remained blinded. Therefore, the risk of major bias is low. Finally, the study focused primarily on early postoperative pain scores and opioid consumption, and did not evaluate multidimensional clinical outcomes – such as the incidence of chronic postsurgical pain, patient-reported recovery quality, and functional recovery metrics.

## 5. Conclusion

In summary, this study indicates that LB combined with TPVB, when used in postoperative analgesia of patients undergoing single-port thoracoscopic lung resection, does not exhibit superior clinical efficacy compared to traditional ropivacaine. Moreover, under the current technical conditions, the overall efficacy of LB combined with TPVB remains limited. In the future, clinical application strategies should be further optimized based on the pharmacological characteristics of LB. For example, it can be used in combination with plain bupivacaine, ultrasound-guided multi-point injection technology can be adopted to improve drug distribution, the dose-effect relationship can be further clarified, and high-quality, large-sample clinical studies can be conducted targeting different types of surgical pain and block methods. Thus, the actual status and value of LB in regional analgesia can be more comprehensively evaluated.

## Data Availability

The full trial protocol and all data that validate the findings of this study are accessible from the corresponding author upon reasonable request.
